# Effects of oestrogens and anti-oestrogens on normal breast tissue from women bearing *BRCA1* and *BRCA2* mutations

**DOI:** 10.1038/sj.bjc.6603042

**Published:** 2006-03-14

**Authors:** M Bramley, R B Clarke, A Howell, D G R Evans, T Armer, A D Baildam, E Anderson

**Affiliations:** 1 Department of Surgery, Christie Hospital NHS Trust, Manchester M20 4BX, USA; 2 Clinical Research Department, Christie Hospital NHS Trust, Manchester M20 4BX, USA; 3 CR-UK Department of Medical Oncology, Christie Hospital NHS Trust, Manchester M20 4BX, USA; 4 University Department of Medical Genetics and Regional Genetics Service, St Mary's Hospital, Manchester M13 0JH, USA

**Keywords:** breast epithelium, oestradiol, tamoxifen, fulvestrant, proliferation, steroid receptors

## Abstract

There is considerable interest in whether anti-oestrogens can be used to prevent breast cancer in women bearing mutations in the *BRCA1* and *BRCA2* genes. The effects of oestradiol (E_2_), tamoxifen (TAM) and fulvestrant (FUL) on proliferation and steroid receptor expression were assessed in normal breast epithelium taken from women at varying risks of breast cancer and implanted into athymic nude mice, which were treated with E_2_ in the presence and absence of TAM or FUL. Tissue samples were taken at various time points thereafter for assessment of proliferative activity and expression of oestrogen and progesterone receptors (ER*α* and PgR) by immunohistochemistry. Oestradiol increased proliferation in the breast epithelium from women carrying mutations in the *BRCA1/2* genes, those otherwise at increased risk and those at population risk of breast cancer. This increase was reduced by both TAM and FUL in all risk groups. In the absence of E_2_, PgR expression was reduced in all risk groups but significantly more so in the *BRCA*-mutated groups. Subsequent E_2_ treatment caused a rapid, complete induction of PgR expression in the population-risk group but not in the high-risk or *BRCA*-mutated groups in which PgR induction was significantly delayed. These data suggest that the mechanisms by which E_2_ induces breast epithelial PgR expression are impaired in *BRCA1/2* mutation carriers, whereas those regulating proliferation remain intact. We conclude that early anti-oestrogen treatment should prevent breast cancer in very high-risk women.

Approximately 5% of all cases of breast cancer are associated with inherited predisposition syndromes and about half of these are attributable to mutations in the *BRCA1* and *BRCA2* genes (Hopper, 2001). Carriers of mutations in the *BRCA1* and *BRCA2* genes have up to a 90% lifetime risk of developing breast cancer (Rebbeck, 2002) and effective preventative strategies are required for these women.

A number of trials have now shown that endocrine agents such as tamoxifen (TAM) or raloxifene can prevent breast cancer in women at increased risk of the disease (Cuzick *et al*, 2003). The question arises as to whether agents such as these can be used for prevention specifically in women bearing *BRCA1* or *BRCA2* mutations. The prevalence of mutations in the total cohort in the NSABP P1 trial was unknown (Fisher *et al*, 1998) and therefore no conclusion regarding the effectiveness of TAM in mutation carriers can be made from this study. It has been suggested that endocrine agents such as TAM will not be effective in preventing breast cancer in *BRCA1* mutation carriers because the majority of tumours (up to 80%) arising in these women are oestrogen receptor *α* (ER*α*) negative, have a high mitotic rate and are of high histological grade (Lakhani, 1999; Lakhani *et al*, 2002). All these features are associated with resistance to endocrine therapy and it is clear from the overview that TAM and raloxifene reduce the incidence of ER*α*-positive but not -negative tumours (Cuzick *et al*, 2003). Against these, however, are data showing that incidence of breast cancer in *BRCA1* and *BRCA2* mutation carriers is altered by endocrine risk modifiers such as pregnancy (Rebbeck *et al*, 2001). Moreover, it is becoming clear that prophylactic oophorectomy not only reduces the incidence of ovarian cancer in mutation carriers but also that of breast cancer (Rebbeck *et al*, 2002). Finally, it has been shown that both oophorectomy and adjuvant TAM treatment reduce the risk of contralateral breast cancer in mutation carriers (Narod *et al*, 2000; Metcalfe *et al*, 2004). This raises the possibility that the initial stages of tumour formation in *BRCA1* and *BRCA2* mutation carriers are hormone dependent, in which case early endocrine therapy might be an effective prevention strategy. This possibility is not addressed by existing trials such as the one run by the NSABP because the effects of TAM became evident after a relatively short follow-up period, suggesting that the anti-oestrogen was preventing progression of existing subclinical lesions rather than inhibiting *de novo* tumorigenesis (Fisher *et al*, 1998).

Significant advances have been made towards understanding the mechanisms by which *BRCA1* and *BRCA2* act. The products of both genes are large, ubiquitously expressed proteins that have fundamental roles in protecting and repairing the genome (Deng and Scott, 2000; Kerr and Ashworth, 2001; Venkitaraman, 2001). The prevailing view on the mechanism by which heterozygous mutations in the *BRCA1* and *BRCA2* genes predispose to cancer is that they provide an unstable genetic environment in which further mutations leading to tumorigenesis are more likely to occur (Deng and Scott, 2000). However, this does not explain why *BRCA1* or *BRCA2* mutations predispose so specifically to breast and ovarian cancer nor why *BRCA1* or *BRCA2* insufficiency can have profound effects on processes such as proliferation and differentiation. For example, global deletion of either gene in mice leads to embryonic lethality due to defects in proliferation and/or differentiation as well as genetic instability (Deng and Scott, 2000; Deng and Brodie, 2001). Conditional mutation of *BRCA1* in the mammary epithelium results in abnormal gland formation and increased apoptosis (Xu *et al*, 1999; Brown *et al*, 2002). Both *BRCA1* and *BRCA2* are expressed in tissues undergoing rapid proliferation prior to undergoing terminal differentiation including the pregnant mammary gland (Chodosh, 1998). In addition, *in vitro* studies indicate that *BRCA1*, in particular, interacts with and represses both nuclear and nongenomic ER*α* signalling pathways (Fan *et al*, 1999; Fan *et al*, 2001; Razandi *et al*, 2004). These data raise the possibility that proliferation and expression of oestrogen-regulated genes are altered in oestrogen target tissues such as the mammary gland. The aims of the present study were two-fold: first, to determine whether *BRCA1* or *BRCA2* haploinsufficiency altered the oestrogen sensitivity of normal breast epithelium in terms of proliferation and induction of oestrogen-induced genes. The second aim was to find out whether tamoxifen, a selective oestrogen receptor modulator (SERM) and fulvestrant, an anti-oestrogen devoid of agonist activity, were able to abrogate the proliferative effects of E_2_ on normal breast tissue from women carrying *BRCA1* and *BRCA2* mutations or who were, otherwise, at increased risk of developing breast cancer. To do this, we used a model of normal human breast tissue implanted into athymic nude mice where it was treated with oestradiol (E_2_) and/or anti-oestrogens. We show that epithelial proliferation in breast tissue from women with *BRCA1* and *BRCA2* mutations or who are otherwise at high risk is stimulated by oestradiol to the same extent to that in tissue taken from women at population risk of breast cancer. The effects of E_2_ on proliferation could be reduced by the addition of TAM or fulvestrant in all three risk groups. However, induction of progesterone receptor expression by E_2_ was impaired in the tissue taken from *BRCA1* and *BRCA2* carriers compared to that taken from women at population risk of breast cancer.

## MATERIALS AND METHODS

### Subjects

Breast tissue was obtained from women at the population risk of breast cancer (*n*=22) at surgery for removal of a fibroadenoma as described previously (Laidlaw *et al*, 1995). ‘Mutated’ tissue was obtained at prophylactic mastectomy from women with documented linkage to or mutations in the *BRCA1* (*n*=5) or *BRCA2* genes (*n*=3). In the case of *BRCA1*, one subject was shown to be a mutation carrier through linkage analysis, but was not sequenced, whereas the other four were characterised by direct sequencing which identified the following mutations: 185delAG, 1294del40, 502del5 and 2799delAA. The three subjects with *BRCA2* mutations were related and direct sequencing confirmed that they had the same lesion, 6819delTG. ‘Increased-risk’ tissue was taken at either prophylactic mastectomy or biopsy in women judged to be at increased risk of breast cancer either by family history or a previous history of cancer (*n*=18). Of these, 12 were undergoing bilateral prophylactic mastectomy because their family history indicated a lifetime risk of breast cancer greater than one in four, three were undergoing prophylactic removal of an uninvolved breast contralateral to one containing tumour and in three cases, tissue was obtained at biopsy from women with a more moderate family history calculated to put them at a lifetime risk of one in six or greater. The three groups (population risk, mutation carriers and high risk) of women were well matched for age at operation and for endocrine breast cancer risk factors such as age at menarche, parity and age at first full-term pregnancy (see Table 1). The study was approved by the Ethics Committee of the South Manchester Hospitals and all subjects gave informed consent for the use of their normal tissue in research studies. In all cases, the tissue used in the implantation studies was examined by an experienced histopathologist (Dr J Coyne) to confirm the presence of normal lobules only. Tissue samples from nine of the 22 population-risk subjects, seven of the eight samples of the ‘mutated’ tissue and 12 of the 18 ‘increased-risk’ samples were used for the implantation studies.

### Implantation of breast tissue into athymic nude mice

All the following procedures were carried out under the Animals (Scientific Procedures) Act of 1986 and complied with UKCCCR Guidelines regarding the ethical use of animals. Implantation of human breast tissue into athymic nude mice was performed as described previously (Laidlaw *et al*, 1995). Each sample of breast tissue was divided into small pieces (2 × 2 × 1 mm), eight of which were implanted subcutaneously (s.c.) into each of at least 16 intact female adult (9–10 weeks old) nu/nu, balb/c mice. At 2 weeks after tissue implantation, control silastic pellets were inserted s.c. into four of the mice, whereas two groups of six mice received 0.5 or 2.0 mg E_2_-silastic pellets prepared as described previously (Laidlaw *et al*, 1995). After 1 week, the two groups of E_2_-treated mice were subdivided such that two mice received injections of the partial agonist anti-oestrogen TAM (1 mg mouse^−1^ day^−1^ in peanut oil s.c.), two received the specific anti-oestrogen fulvestrant (ICI 182 780; 5 mg mouse^−1^ week^−1^ s.c. in castor oil) and two the appropriate vehicle control (peanut or castor oil). These doses were chosen because they had been shown to inhibit the growth of MCF-7 human breast tumours in athymic nude mice (Osborne *et al*, 1995) and treatments were continued for 2 weeks. Two tissue samples were removed from each of the mice 2, 3, 4 and 5 weeks after implantation, fixed in 4% buffered formalin overnight at 4°C, then embedded in paraffin wax. Blood was taken from the mice at the conclusion of each experiment and serum E_2_ levels were assayed by RIA as described (Laidlaw *et al*, 1995). The median serum E_2_ concentration in the mice treated with control silastic pellets was 107 pM with an interquartile (IQ) range of 74–214 pM (*n*=100), those treated with 0.5 mg E_2_ pellets had a final median concentration of 386 pM (IQ range 268–745 pM; *n*=59), whereas those treated with 2 mg E_2_ pellets achieved a final median serum concentration of 1050 pM (IQ range 785–1660 pM; *n*=57).

### Immunohistochemical assessment of proliferation and steroid receptor expression

Proliferative activity was assessed by immunohistochemistry (IHC) using the mouse monoclonal antibody MIB-1 (Coulter Ltd, UK) raised against the Ki67 proliferation-associated antigen. Expression of ER*α* and PgR was determined by IHC using a mouse monoclonal anti-ER*α* antibody (clone ID5, Dako Ltd, UK) and a rat monoclonal anti-PR antibody (clone KD68, Abbott Laboratories, UK), respectively. Microwave antigen retrieval methods and dilutions were as described previously (Clarke *et al*, 1997a). Antibody binding was detected indirectly using the appropriate biotinylated second antibodies, a peroxidase-conjugated avidin–biotin complex (ABC Elite, Vector Laboratories, UK) and diaminobenzidine as the chromogen. Quantitation of immunostaining was carried out on a light microscope and was restricted to the epithelial cells of the terminal duct lobulo-alveolar units. Areas to be counted were selected out of focus at low power and then complete high-power fields were scored. At least 1000 epithelial cells were scored per sample and the number of labelled cells was expressed as a percentage of the total cells counted. The intensity of staining was not assessed.

### Statistical methods

Throughout, the data are presented as medians together with their respective interquartile ranges (IQRs). Overall changes across treatment or risk groups were analysed using the Kruskal–Wallis nonparametric, one-way analysis of variance. If this proved significant at the 5% level, then pairwise comparisons were made using the nonparametric Mann–Whitney *U*-test.

## RESULTS

### Oestradiol stimulates breast epithelial cell proliferative activity in all risk groups

**Figure 1:**
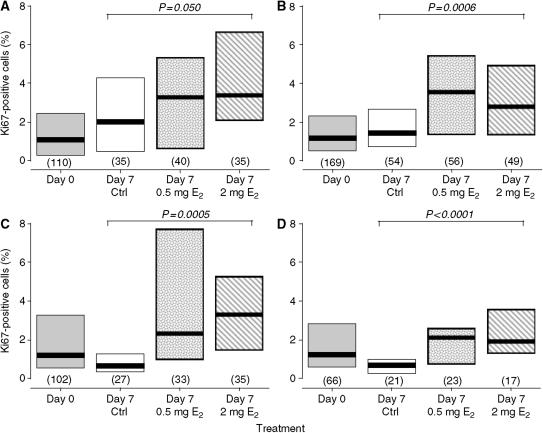
Proliferative activity of normal breast epithelium taken from women at population risk of breast cancer (**A**), those at high risk (**B**) All *BRCA1* or *BRCA2* mutation carriers (**C**) and *BRCA1* mutation carriers only (**D**), which had been implanted into athymic nude mice before (day 0) and after 1 week's treatment with control, 0.5 mg E_2_ or 2.0 mg E_2_-silastic pellets. The thick horizontal lines indicate the median values as do the figures above the columns indicating the IQRs. The numbers in parentheses are the numbers of samples available for analysis in each group and the *P-*values indicate the significance of the differences across the treatment groups by Kruskal–Wallis nonparametric analysis of variance. Ctrl=control.

Analysis of the tissue samples, fixed at the time of removal from the patients, revealed no differences in the percentages of breast epithelial cells labelled with the anti-Ki67 antibody between the different risk groups (see Table 2). At 2 weeks after implantation into the mice and before treatment commenced, epithelial Ki67 expression decreased to approximately 1% and, again, there were no significant differences between the samples taken from the different risk groups (see Table 3 and Figure 1). Figure 1 shows the proliferative activity of the tissue samples from the different risk groups taken before (day 0) and 1 week (day 7) after the start of E_2_ or control treatment. In the absence of E_2_, epithelial Ki67 expression in the tissue samples from the population- and high-risk tissue groups remained at the levels seen before treatment (Figure 1A and B). The median Ki67LI in the tissue samples from the ‘All mutations’ group (Figure 1C) decreased although not significantly so. However, separate analysis of the samples from women bearing *BRCA1* mutations (Figure 1D) indicated that, in the absence of E_2_, the epithelial Ki67LI decreased significantly to a median of 0.7% (IQR=0.4–1.0; *n*=21; *P*=0.009 by Mann–Whitney *U*-test). The reasons for this decline in proliferative activity in the tissue taken from women carrying *BRCA1* mutations compared to that from women at population risk are not clear at present, but it may be due simply to the variability in the data from the latter group. Figure 1 also shows the breast epithelial Ki67LIs in each of the risk groups after 1 week's treatment with 0.5 and 2 mg E_2_. In the population-risk group, there was an increase in the Ki67LI after treatment with the 0.5 mg E_2_ pellet although this was not statistically significant. In the other three risk groups, the Ki67LI increased significantly after treatment with the low-dose E_2_ pellet (*P*<0.001 by Mann–Whitney *U*-test) compared to the appropriate untreated control. Treatment with the high-dose (2 mg) E_2_ pellet (Figure 1C) significantly increased the median Ki67LIs in all four of the risk groups (*P*<0.001 by Mann–Whitney *U*-test) when compared to the corresponding control values.

### Induction of progesterone receptor expression by oestradiol is impaired in breast tissue from *BRCA* mutation carriers

**Figure 2:**
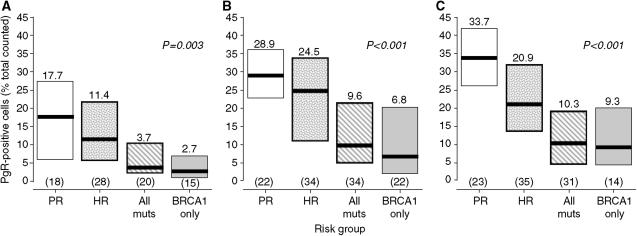
Progesterone receptor expression in normal breast epithelium taken from women at population risk of breast cancer, those at high risk and those carrying *BRCA1* and *BRCA2* mutations, which had been implanted into athymic nude mice and treated for 1 week with control, (**A**), 0.5 mg E_2_ (**B**) or 2.0 mg E_2_ (**C**)-silastic pellets. The thick horizontal lines indicate the median values as do the figures above the columns indicating the IQRs. The numbers in parentheses are the numbers of samples available for analysis in each group and the *P-*values indicate the significance of the differences across the risk groups by Kruskal–Wallis nonparametric analysis of variance. PgR=progesterone receptor; PR=population risk; HR=high risk; All muts=all mutations; BRCA1 only=*BRCA1* mutation carriers only.

There were no significant differences in the levels of breast epithelial cell ER*α* and PgR expression between the risk groups at the time tissue was removed from the women (Table 2). However, 14 days after implantation into untreated mice, there was a significant decrease in the percentage of PgR-positive cells in all risk groups (Table 3). This was most pronounced in the tissue samples from women bearing *BRCA1* mutations and resulted in the percentage of PgR-positive cells being approximately four-fold lower than that of the population-risk group (*P*<0.001 by Mann–Whitney *U*-test). Figure 2 shows the effects of 1 week's treatment with control, low- and high-dose E_2_-silastic pellets on PgR expression in breast epithelial cells. Levels of PgR expression were not altered in the control-treated samples (Figure 2A), and the differences between the risk groups were maintained. Figure 2B shows that treatment with the low-dose E_2_ pellet increased epithelial cell PgR expression in samples from all the risk groups. However, the responses to low-dose E_2_ in the All mutations and *BRCA1* groups were attenuated such that the percentages of PgR-positive epithelial cells were increased only to 9.6% (IQR=4.9–21.3) and 6.8% (IQR=2.1–20.4), respectively. These increases in PgR expression in the samples from the All mutations and *BRCA1* groups almost, but not quite, reached statistical significance when compared to the corresponding control-treated samples (*P*=0.051 and 0.07, respectively, by Mann–Whitney *U*-test). Expression of the PgR in breast epithelium from all the risk groups was also induced by 1 week's treatment with high-dose E_2_ (Figure 2C). However, the increase in the samples from the high-risk group did not reach the same level as that in the population-risk samples (*P*=0.002 by Mann–Whitney *U*-test). The responses in the All Mutations and *BRCA1* groups were diminished considerably in that they reached medians of only 10.3% (IQR=4.6–19.2) and 9.3% (IQR=4.4–20.0), respectively (*P*=0.062 and 0.012 *vs* corresponding control-treated samples by Mann–Whitney *U*-test).

**Figure 3:**
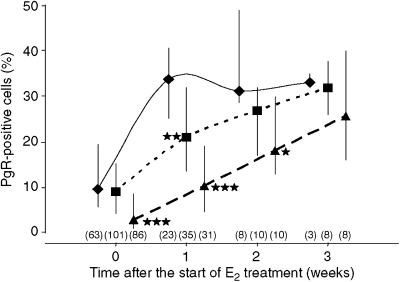
The effects of E_2_ treatment over time on progesterone receptor expression in breast epithelium from women at population risk of breast cancer (⧫) compared to that from women at high risk (▪) and those carrying *BRCA1* and *BRCA2* mutations (▴). The median values are represented by the solid symbols, whereas the error bars indicate the IQRs and the numbers in parentheses are the samples available for analysis in each group. ^*^P<0.05, ^**^P<0.01 and ^***^P<0.001 compared to the population risk group by Mann–Whitney *U*-test. PgR=progesterone receptor.

The effects of the high-dose E_2_ pellet on PgR expression in human breast epithelium at all time points after the start of treatment are shown in Figure 3. In the tissue samples from the population-risk group, PgR expression was maximal after 1 week of treatment with the high-dose E_2_ pellet and this level was maintained for the remainder of the experimental period. In contrast, the response in the All mutations group was attenuated such that percentages of PgR-positive cells in samples from this group were significantly lower than those of the population-risk group not only before the start of treatment but also for 2 weeks afterwards (*P*<0.001 by Mann–Whitney *U*-test at each time point). By the final time point, the median percentage of PgR-positive cells in samples from the All Mutations group had increased such that it approximated to that of the population-risk group. The effects of E_2_ on PgR expression in tissue samples from the *BRCA1* group could not be examined separately due to the small number of tissue samples available for analysis at the later time points. Examination of the effects of E_2_ on PgR expression in the tissue samples from the high-risk group of women confirmed the results shown above in that the percentage of PgR-positive epithelial cells in this group after 1 week of high-dose E_2_ treatment was significantly lower than that of the population-risk group but thereafter, there were no significant differences between the two groups.

### Tamoxifen and fulvestrant reverse the effects of E_2_ on breast epithelial cell proliferative activity in all risk groups

**Figure 4:**
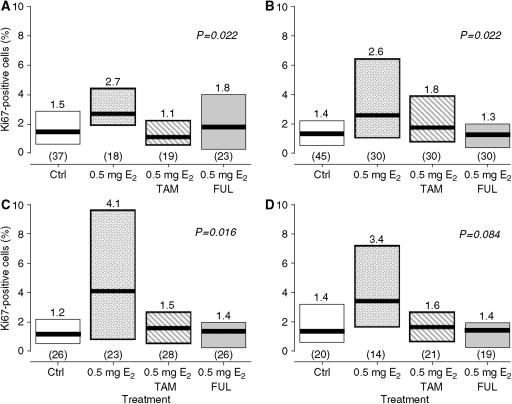
The proliferative activity of normal breast epithelium taken from women at population risk of breast cancer (**A**), those at high risk (**B**), those carrying *BRCA1* and *BRCA2* mutations (**C**) and *BRCA1* mutation carriers only (**D**) after implantation into athymic nude mice and treatment with 0.5 mg E_2_ for 1 week followed by 2 weeks of E_2_ combined with either TAM or fulvestrant. The thick horizontal lines indicate the medians as do the numbers on top of the columns indicating the IQRs. The numbers in parentheses are the numbers of samples available for analysis in each treatment group and the *P-*values indicate the significance of the differences across the treatment groups by the Kruskal–Wallis nonparametric analysis of variance. Ctrl=control; 0.5 mg E_2_=treatment with 0.5 mg E_2_-silastic pellets; TAM=tamoxifen (1 mg mouse^−1^ day^−1^); FUL=fulvestrant (5 mg mouse^−1^ week^−1^).

Figure 4 shows Ki67 expression in normal breast tissue samples taken from the mice after treatment with 0.5 mg E_2_ for 1 week followed by a further 2 weeks of treatment in which the E_2_ was combined with either TAM or fulvestrant. An additional group of mice were treated with 0.5 mg E_2_ only, whereas the negative control group received vehicle alone. Treatment with 0.5 mg E_2_ was sufficient to increase Ki67 expression in the population-risk, high- risk and All mutations groups (Figures 4A, B and C; *P*<0.01 by Mann–Whitney *U*-test compared to the appropriate untreated control for each risk group). Addition of TAM at a dose of 1 mg mouse^−1^ day^−1^ reduced expression of Ki67 in these three risk groups compared to 0.5 mg E_2_ alone (*P*<0.05 by Mann–Whitney *U*-test in each risk group). The percentage of Ki67-positive cells was also reduced by the addition of fulvestrant (5 mg mouse^−1^ week^−1^) in the population risk, increased=risk and All Mutations groups compared to 0.5 mg E_2_ alone (Figure 4A, B and C; *P*<0.05 by Mann–Whitney *U*-test in each risk group). Both TAM and fulvestrant, when added to 0.5 mg E_2_, reduced the median percentages of Ki67-positive breast epithelial cells to the levels seen in untreated controls in the population-risk, increased-risk and all mutations groups (no significant difference when compared to controls by Mann–Whitney *U*-test in each risk group). Separate analysis of the tissue samples from women bearing *BRCA1* mutations (Figure 4D) showed that the effects of 0.5 mg E_2_ in the absence and presence of TAM or fulvestrant did not quite reach statistical significance (*P*=0.084 by Kruskal–Wallis test) although the pattern of changes reflected those seen in the other risk groups.

The ability of TAM and fulvestrant to reverse the effects of high-dose (2 mg) E_2_ treatment on breast epithelial cell Ki67 expression was also investigated (data not shown). Both anti-oestrogens could reduce the effects of E_2_ on proliferation in all the risk groups but their effects were rather more variable presumably because the serum drug levels achieved with the above dosing schedules were not sufficient to overcome the high plasma E_2_ levels reached after treatment with the 2 mg E_2_ pellet.

## DISCUSSION

The effects of heterozygous mutations in the *BRCA1* or *BRCA2* breast cancer-predisposing genes on the endocrine sensitivity of human breast epithelial cells are unknown. It is important to determine what these effects may be because of the increasing use of endocrine agents such as TAM for breast cancer prevention in *BRCA1* and *BRCA2* mutation carriers. Although the BRCA1 and BRCA2 proteins play critical roles in DNA damage repair (Brodie and Deng, 2001; Deng and Brodie, 2001; Kerr and Ashworth, 2001), *in vitro* studies suggest that the *BRCA1* gene product can also interact with and suppress the activity of the ER*α* (Fan *et al*, 1999; Fan *et al*, 2001). As E_2_ is the major steroid mitogen for the human breast epithelium acting via the ER*α*, it might be predicted that *BRCA1* and, possibly, *BRCA2* haploinsufficiency would result in an enhanced proliferative response to E_2_. The present data show that this is not the case as proliferation of breast epithelium from mutation carriers in response to E_2_ treatment does not differ significantly from that of tissue from women at population risk of the disease. Secondly, it might be predicted that *BRCA1* and *BRCA2* haploinsufficiency would confer anti-oestrogen resistance to the breast epithelium. Again, we show that this is not the case as both the SERM TAM and the specific anti-oestrogen fulvestrant could reverse the effects of E_2_ on proliferation. Both anti-oestrogens also reversed the proliferative effects of E_2_ in tissue from women at high risk of breast cancer because of their family history. These findings lead us to suggest that early intervention to antagonise the effects of E_2_ or reduce serum E_2_ levels would be effective for the prevention of breast cancer in *BRCA1* or *BRCA2* mutation carriers. As both the BRCA1 and BRCA2 proteins are involved in postreplicative DNA repair (Brodie and Deng, 2001; Deng and Brodie, 2001; Kerr and Ashworth, 2001), reducing the proliferative activity of the breast luminal epithelial cell population from which most tumours are derived should reduce the number of opportunities for replication errors to occur. The propagation of potentially transforming mutations might also be reduced.

Although there are no apparent alterations in the proliferative activity of the tissue from *BRCA1* and *BRCA2* mutation carriers, we have been able to detect a defect in the kinetics of PgR induction by E_2_. As far as we are aware, this is one of the first reports of a biological phenotype associated with heterozygous mutations in the *BRCA1* and *BRCA2* genes, and complements the recent reports of an effect on PgR expression by Mote *et al* (2004). The delayed PgR response to E_2_ in the mutation carriers is paradoxical in the face of the *in vitro* evidence suggesting that the *BRCA1* gene product is a repressor of both nuclear and nongenomic mechanisms of ER*α* action (Fan *et al*, 1999, 2001; Razandi *et al*, 2004). However, some of these experiments used reporter constructs containing a consensus oestrogen response element (ERE) from the vitellogenin gene to examine the effects of the BRCA1 protein on ER*α* transcriptional activity (Fan *et al*, 1999), whereas others have shown that overexpression of BRCA1 abrogates the effects of E_2_ on expression of the oestrogen-induced genes, pS2 and cathepsin D (Fan *et al*, 2001). The PgR gene promoter does not contain a complete ERE but it does have ERE half sites adjacent to those for Sp-1 (Petz and Nardulli, 2000), and it is possible that the BRCA1 protein has significantly different effects at this site in human breast epithelium *in vivo*. Alternatively, the differences in expression of PgR in response to E_2_ treatment may result from an alteration in the ratio of the A and B isoforms of the PR. Many commercially available antibodies used for PgR IHC fail to detect the B form of the receptor in formalin-fixed, paraffin-embedded tissues (Mote *et al*, 2001). The specificity of the antibody used in the present study is not known at present, but it is probable that it also does not detect PgRB in routinely processed tissue. Therefore, the apparent alteration in PgR induction following E_2_ treatment of tissue from women with *BRCA* mutations may reflect an alteration in the PgRA to PgRB ratio such that PgRB expression is increased at the expense of PgRA. One way of confirming this would be to use more specific antibodies to determine the ratios of the two PgR isoforms in the breast epithelium from the mutation carriers compared to those from subjects at population risk of breast cancer. This approach has been used by Mote *et al* (2004) who have shown that not only is the ratio of PgRA to PgRB altered in favour of PgRA in normal breast tissue taken from *BRCA1* and *BRCA2* mutation carriers but also that expression of both isoforms is markedly reduced compared to tissue from normal-risk women. This suggests that the reduced levels of PgR expression seen in the mutation carriers in the present study is due to a reduction in both PgR isoforms. Finally, we have shown that luminal epithelial cells that proliferate in response to E_2_ do not contain the ER*α*, whereas PgR is expressed only in ER*α*-positive cells (Clarke *et al*, 1997b). Clearly, BRCA1 would act as a coregulator of ER*α* activity only in cells that contain the receptor; therefore, we might expect BRCA1 to be involved directly in the control of PgR expression but not proliferation and this is supported by the present data. It will be important to determine exactly how the *BRCA1* and *BRCA2* gene products contribute to the control of PgR expression in the human breast epithelium. The effects of E_2_ on PgR expression in tissue from the high-risk group of women also appeared to be altered although the differences between this group and the population risk group just failed to reach significance. This may reflect the fact that many of the women in this risk group were given a probability of developing breast cancer of at least one in four based on their family history. Accordingly, one in four of these women could be carrying *BRCA1* and *BRCA2* mutations, whereas the others will be at population risk of the disease. This would dilute the alteration in the effects of E_2_ on PgR expression associated with *BRCA* gene mutations in this group.

A potential criticism of our line of thinking could be that there is no haploinsufficient effect of a *BRCA1* or *BRCA2* mutation, and that anti-oestrogen treatment functions only as a secondary preventer of breast cancer. Given that primary prevention of breast cancer would have to take place at least 6 and possibly 10 years prior to a clinically apparent breast tumour being detected, none of the prevention trials and particularly not the NSABP trial (King *et al*, 2001) would have been powered to detect a primary preventive role. There is nonetheless evidence from *BRCA1* mutation carriers that TAM may be effective in long-term prevention of second primary cancers and part of this is likely to be due to a primary preventive role. This is because the level of reduction seen would not be consistent with secondary prevention of what are mostly ER-negative tumours (Narod *et al*, 2000; Metcalfe *et al*, 2004). That primary prevention of breast cancer in *BRCA* carriers occurs by reducing oestrogen stimulation of the breast is now beyond dispute. Oophorectomy prior to the menopause substantially reduces the risks of breast cancer (Rebbeck *et al*, 2001, 2002). Even though *BRCA1* cancers are thought to derive from basal cells that are ER negative, these cells are still under the influence of neighbouring ER-positive epithelial cells. Reducing the growth-inducing signals of these cells will potentially reduce proliferation of the basal cells and reduce the chances of introducing mutations during cell division by naturally occurring replication error. Even if there is no heterozygous effect on the cell of carrying a *BRCA* mutation, anti-oestrogen treatment is still likely to be effective in primary prevention by this mechanism. Yet, there may well be a haploinsufficient effect. We have shown that there does appear to be a difference between *BRCA1-*mutated epithelial cells and controls. Moreover, Kote-Jarai *et al* (2004) have shown that fibroblasts from *BRCA1* mutation carriers can be distinguished from controls in terms of their response to radiation damage in microarray analysis. The elusive haploinsufficient effect and a functional assay for the heterozygous state may not be too far away.

In summary, we have shown that E_2_-stimulated proliferation in breast epithelium taken from women bearing heterozygous mutations in the *BRCA1* and *BRCA2* genes can be reversed by both a SERM (TAM) and an anti-oestrogen (fulvestrant) that is without agonist effects. We have also shown that altered expression of PgR is a phenotype associated with mutations in the *BRCA1* and *BRCA2* genes. We conclude that early anti-oestrogen treatment should be effective for the prevention of breast cancer in high-risk women.

## Figures and Tables

**Table 1:** Characteristics of the subjects from whom tissue was taken for analysis of the effects of oestradiol and anti-oestrogens on proliferation and steroid receptor expression in normal breast epithelium

	**Population risk**	**Mutated**	**High risk**	
Age at operation (years)				
*All subjects*				
Median	36.5	37	37.5	nsd^a^
Range	16–46	29–53	24–54	
*n*	22	8	18	
				
*Subjects whose tissue was implanted*				
Median	40	39	38.5	nsd
Range	31.5–44	29–53	25–54	
*n*	9	7	12	
				
Age at menarche (years)				
*All subjects*				
Median	13	13	12.5	nsd
Range	10.5–16	11–15	10–16	
*n*	20	7	18	
				
*Subjects whose tissue was implanted*				
Median	12.25	13	12.5	nsd
Range	11.5–16	11–15	11–15	
*n*	9	6	12	
				
Parity				
*All subjects*				
Median	2	2	2	
Range	0–4	0–3	0–7	nsd
*n*	22	8	18	
				
*Subjects whose tissue was implanted*				
Median	2	2	3	nsd
Range	0–4	0–3	0–7	
*n*	9	7	12	
				
Age at FFTP^b^ (years)				
*All subjects*				
Median	22	25.5	21	
Range	17–33	19–35	17–30	nsd
*n*	17	6	15	
				
*Subjects whose tissue was implanted*				
Median	21.5	25.5	21	nsd
Range	18–33	19–35	19–30	
*n*	8	6	11	

Population risk=women at population risk of breast cancer undergoing removal of a fibroadenoma; ‘Mutated’=women carrying mutations in the *BRCA1* and *BRCA2* genes undergoing prophylactic mastectomy; High risk=women at very high risk of breast cancer due to family history also undergoing prophylactic mastectomy or biopsy in women judged to be at increased risk of breast cancer either by family history or a previous history of cancer.

a nsd=no significant difference between the risk groups by Kruskal–Wallis test.

b FFTP=first full-term pregnancy.

**Table 2:** Proliferation (Ki67), oestrogen receptor alpha (ER*α*) and progesterone receptor (PgR) expression in samples of breast epithelium taken from women at population risk of breast cancer undergoing removal of a fibroadenoma, from *BRCA1* or *BRCA2* mutation carriers undergoing prophylactic mastectomy (‘mutated’) or from women at high risk of breast cancer also undergoing prophylactic mastectomy or biopsy in women judged to be at increased risk of breast cancer either by family history or a previous history of cancer

	**Population risk**	**High risk**	***BRCA1*+*BRCA2***	***BRCA1* only**	
*n*	22	18	8	5	
*Ki67 (%)*					
Median	2.3	1.5	2.9	2.9	nsd^b^
IQ range^a^	1.3–8.2	1.1–3.5	0.9–4.0	0.7–4.8	
*ERα (%)*					
Median	27.9	21.6	29.6	27.3	nsd
IQ range	16.9–32.9	17.3–35.8	12.2–35.2	13.2–36.0	
*PgR (%)*					
Median	24.8	20.6	19.5	15.3	nsd
IQ range	16.3–35.6	15.9–26.4	9.4–28.8	4.8–24.7	

The figures represent the proportion of positively stained epithelial cells expressed as a percentage of the total number of cells counted.

a IQ range – interquartile range.

b nsd=no significant difference between the three risk groups by Kruskal–Wallis test.

**Table 3:** Proliferation (Ki67) and progesterone receptor (PgR) expression in samples of human breast epithelium 14 days after being implanted into the athymic nude mice and before the initiation of treatment

	**Population risk**	**High risk**	***BRCA1* + *BRCA2***	***BRCA1* only**	
*Ki67 (%)*					
Median	1.08	1.16	1.21	1.25	nsd^a^
IQ range^b^	0.3–2.45	0.50–2.30	0.57–3.28	0.61–2.84	
*n*^c^	110	169	102	66	
					
*PgR (%)*					
Median	9.60	9.12	2.94	1.99	*P*<0.001^a^
IQ range	5.34–18.48	4.32–15.30	0.96–8.51	0.90–5.04	
*n*	63	101	86	52	

The figures represent positively stained epithelial cells expressed as a percentage of the total number of cells counted.

a Significance of comparison between risk groups by Kruskal–Wallis test; nsd=no significant difference.

b IQ range=inter-quartile range.

c *n*=number of tissue samples in which parameter could be measured.
